# Making Our Hospitals a Safe Workplace: Hazard Identification and Risk Assessment at a Tertiary-Level Public Hospital in Eastern India

**DOI:** 10.7759/cureus.59110

**Published:** 2024-04-26

**Authors:** Biswajeevan Sahoo, Mukunda C Sahoo, Jawahar S Pillai

**Affiliations:** 1 Hospital Administration, All India Institute of Medical Sciences, Bhubaneswar, IND

**Keywords:** risk assessment, occupational safety and health, hospital, hira, risk scoring, safety, hazard

## Abstract

Background: Hospitals are complex places with a large number of employees, patients, furniture, equipment, etc. Healthcare workers (HCWs), patients, or the general public are vulnerable to injuries and illness due to unseen hazards at the workplace. This study aims to identify the hazards and assess the risks at a hospital to ensure safety for HCWs, patients, and the public and generate awareness about the same. It helps in reducing the financial obligation of the institution due to the treatment of illnesses of staff, absenteeism, and service disruption and slows down manpower turnover. Hazard Identification and Risk Assessment (HIRA) helps reduce human errors and promote safe behavior.

Objective: This study aims to identify and study the hazards in a hospital, assess the risks associated with the hazards, and recommend methods to reduce or eliminate the hazards based on the outcomes of the study.

Methodology: An observational study was conducted at a 1000-bed tertiary-level teaching public sector hospital in eastern India. A checklist was used for direct observation, conducting staff interviews, and document reviews. A risk scoring tool was used, and hazards were ranked as per the risk score.

Results: Thirty-eight hazards were identified in the study and classified under the categories of natural, physical, chemical, biological, ergonomic, psychological, and safety. The fire risk and occurrence of cyclones had the highest risk scores.

Conclusions: The study identified hazards through direct observations, record reviews, and staff interviews. These findings can guide the prioritization of areas requiring necessary action in risk reduction, ensuring a safe workplace for healthcare workers (HCWs), patients, and the public. They can also help the institution shift from a reactive approach to a proactive method for HCW safety.

## Introduction

The safety of the public, patients, and staff at the workplace is necessary at all times. Workplace hazards are not easily predicted or visualized. Hospitals undergo periodic modifications with the addition of newer facilities, thereby becoming more susceptible to hazards. A hazard is any potential damage, harm, or adverse health effects on any person or an establishment [1]. It may be in the structure, the process, or the outcome of various activities performed. Hazards can be natural or man-made. Hazards can also be classified as physical, chemical, biological, ergonomic, psychological, and safety-related. Hazard identification is intended to inspect the facilities and processes and identify situations, items, or things that have the potential to cause harm. Hazard identification can be done by collecting information from staff interviews, like faculty, managers, housekeeping staff, security personnel, doctors, nurses, etc. Incident reporting systems, checklist-based facility inspection rounds, process and activity charts, the tracer method for the journey of a patient or staff in the hospital, safety audits, medical record reviews, committee reports, assessment or accreditation reports, incident reports, minutes of meetings, circulars from the heads of institutions, feedback and grievance reports, surveys, logbooks, or complaint book reviews are other methods to identify hazards.

Severity is the seriousness of the potential injury or the consequence due to exposure to any hazards. It can be scaled depending on the extent of damage and its severity. It can be subdivided into human impact, infrastructural impact, financial impact, or any other tangible or intangible damages.

Risk is the possibility or chance that a person will be harmed or suffer an adverse health effect if he/she is exposed to a hazard [2]. The risk applies to disruption of services, financial losses, property loss, or environmental damage due to the hazards. Risk can be reduced through the proactive identification of hazards and the assessment of the risks associated with them.

The likelihood is how likely an accident is to occur and cause harm. This can be understood from past records of similar or related events. Severity is the seriousness of the injury or harm.

Risk assessment is a systematic process of identifying hazards, evaluating any associated risks within a workplace, and then implementing reasonable control measures to remove or reduce them. The purpose of risk assessments is to evaluate the effectiveness and suitability of existing control measures, ensure additional controls are implemented, and prioritize further resource allocation for the same.

Need for hazard identification and risk assessment (HIRA) in hospitals

Hospitals are highly complex facilities equipped with sophisticated equipment, oxygen and gas supply systems, furniture, drugs and consumables, stationery, and departments like registration, billing, out-patient departments, laboratories, radio diagnosis, the central sterile services department (CSSD), kitchen and dietary services, wards, operation theaters (OTs), labor rooms, dialysis units, intensive care units (ICUs), linen and laundry services, biomedical engineering, engineering services, air conditioning systems, electrical units, stores, and purchase divisions, pharmacy units, waste management systems, etc. working in a coordinated manner. This involves a large number of staff working 24 x 7. There are instances of injury among staff in the hospital, for which they undergo necessary treatment and eventually become accustomed to it in the workplace, while the hazard remains unattended. Newly joined staff like doctors, nurses, and interns often face injuries. Further, patients are always new to the hospitals, face difficulties, and are usually more vulnerable to injuries [3,4].

This study was conducted at a 1000-bed tertiary-level teaching institution in eastern India, which has more than 4000 healthcare workers (HCWs). The study setting is a nine-year-old facility with newly established infrastructure, including OTs, ICUs, laboratories, and imaging. HIRA is necessary for the hospital to build a safer environment [5] and generate a sense of responsibility among the staff and trust among the patients who visit the hospital. There is a need to change from a reactive approach to a proactive approach. The institution is situated in eastern India and is an institute of national importance. About 4000 patients visit the hospital every day, with 100 admissions per day, and about 200 patients are treated at the trauma and emergency unit. Thus, the hospital is a major healthcare provider in the region. Natural calamities like cyclones often cause serious disruptions in the supply chain and essential resources like water, electricity, food, medicines, and human resources.

## Materials and methods

This is an observational study conducted for 18 months. The study areas were the hospital block, medical gas plant, electrical substation, air conditioning plant, and waste management complex. The academic block, residential block, and mortuary were excluded for logistics convenience. The Institutional Ethics Committee of All India Institute of Medical Sciences approved the study (IEC/AIIMSBBSR/PGThesis/2019-20/80).

The study was conducted in three phases. The first phase was preparation, knowledge building, and checklist development. The study team underwent training in healthcare risk management by the Consortium of Accredited Healthcare Organizations for 15 days. A checklist was developed by the study team and validated by external experts in the department of hospital administration. The second phase was direct observation, where facility rounds were conducted in the hospital block as per the checklist [6,7]. The basement, waiting areas, registration areas, staircases, wards, ICUs, OTs, labor complex, radiodiagnosis, central laboratories, central sterile supply services, hemolysis unit, blood bank, central pharmacy, medical records department, and hospital kitchen were included along with other areas mentioned earlier. A list of hazards was prepared. Review meetings were conducted with the faculty members of the department of hospital administration and external resource persons having expertise in healthcare administration. A risk-scoring tool was used for scoring the hazards identified in the study. A scale of 1-3 was used, where 3 was high risk, 2 was medium risk, and 1 was low risk. A score of zero was assigned for the hazards that were not applicable in certain areas of observation. The third phase was record review, staff interviews, and risk scoring. It included a document review of complaint books, registers, and logbooks of the civil, electrical, air conditioning, and medical gas supply units; incident report registers of the fire safety and security departments at wards, ICUs, and OTs; complaints received at the department of hospital administration and the office of the medical superintendent through letters and email communications; reports of various quality council committees, viz. hospital infection control committee and waste management committee, medical audit and sentinel event monitoring committee, medication safety committee, fire safety committee, and communication committee. Based on these categories of hazards and the findings of the record review, a questionnaire was developed for interviewing the healthcare staff. It was validated by the faculty members of the department of hospital administration in the review meetings conducted at the department. The HCWs at supervisory and managerial levels were selected for the staff interview. Staff at supervisory and managerial levels with a minimum experience of five years were identified for interviews. The sample size was 67 at 95% CI, and the margin of error was 5%.

Risk scoring

The presence of the hazard in the area of observation was marked "1," and its absence was marked "0." The mean score for the presence of the hazard was calculated. Risk scoring was done on a scale of 1 (low risk) to 3 (high risk) for the identified hazards for both direct observation and with findings from staff interviews. The hazards were further correlated with the record review. A score of zero was given to the hazard, which had no supportive records. Similarly, a score of 1 was given for hazards with complaints mentioned in the registers or logbooks. A score of 2 for medium risk and 3 for high risk was given to the hazards, which were identified with incident reports, hospital infection control team reports, and Kayakalp (a sanitation and quality improvement scheme by the government of India). Review meeting minutes, reports from the fire safety team, committee reports, and email communications received at the MS office and department of hospital administration control room. Finally, the average risk score was calculated, and the identified hazards were ranked in descending order of the risk score.

## Results

**Figure 1:**
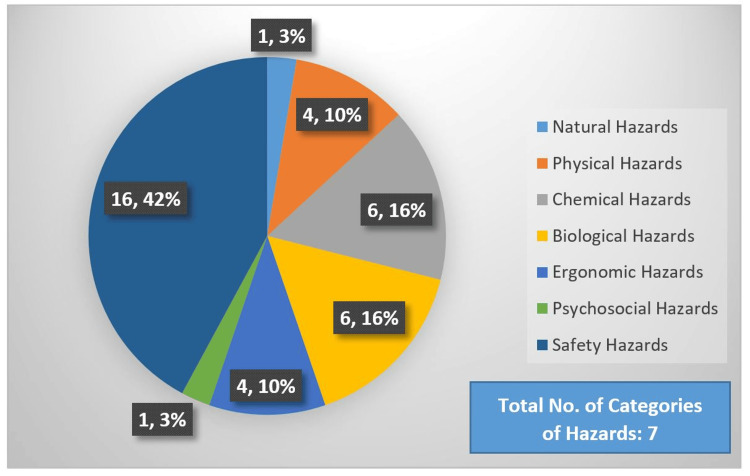
Categories of hazards identified (number, percentage) Seven categories of hazards were identified

A total of 38 hazards were identified. The safety hazards were among the largest in number (41% of the total hazards) (Figure 1).

The top 12 hazards, having a risk score of more than 2, were inadequate staff training in fire safety (H27), damage due to the occurrence of the cyclone (H1), non-availability of appropriate fire extinguishers in the units (H23), non-availability of standard operating procedures (SOP) of fire safety (H26), non-availability of the fire signage and evacuation maps (H25), non-functional fire detection and protection system (H24), non-availability of the preventive maintenance program of furniture (H36) and equipment (H35), non-availability of information, education, and communication (IEC) materials and SOP for waste management (H16) in some of the areas, non-availability of patient shifting aids (H21), non-availability of periodic water filter cleaning system (H12), and non-availability of safety devices on wheelchairs, stretchers, and beds (H37) (Table 1).

**Table 1:** List of hazards with risk score The hazards identified are numbered H (no.) and are ranked in descending order of risk score DO: direct observation, SI: staff interview, RR: record review, SOP: standard operating procedure, IEC: information, education, and communication, BMW: biomedical waste, MSDS: material safety data sheet, PPE: personal protective equipment, CSSD: central sterile supply department, MGPS: marine growth prevention system

Rank	Hazard no.	Hazard identified	Category	Average score from DO	Average score from SI	Score from RR	Final risk score
1	H27	Staff are not trained in fire safety	Safety	2.38	3.0	3	2.79
2	H1	Damage or injury due to the occurrence of natural phenomena - cyclones	Natural	2.53	2.81	3	2.78
3	H23	Non-availability of appropriate fire extinguishers in the unit	Safety	2.63	2.54	3	2.72
4	H26	Non-availability of SOP for fire safety	Safety	2.53	2.57	3	2.70
5	H25	Non-availability of fire safety signage and evacuation maps	Safety	2.84	2.18	3	2.67
6	H24	Fire safety installation is non-functional - fire detection and alarm system, hydrant system	Safety	2.68	1.93	3	2.54
7	H36	Non-availability of preventive maintenance program for hospital furniture	Safety	2.06	2.42	3	2.49
8	H35	Non-availability of preventive maintenance program of equipment	Safety	2.06	2.03	3	2.36
9	H16	Non-availability of IEC and SOP for general waste and BMW management	Biological	1.69	2.03	3	2.24
10	H21	Non-availability of patient shifting/transfer aids like slide boards	Ergonomic	1.75	1.88	3	2.18
11	H12	Non-availability of periodic water filter cleaning mechanism	Biological	1.63	1.72	3	2.11
12	H37	Non-availability of safety devices on wheelchairs/stretchers/beds	Safety	2.56	1.78	2	2.11
13	H8	Non-availability of spill kit	Chemical	1.60	1.93	2	1.84
14	H11	Concerned staff not trained in handling chemicals, spill management, and accidental exposure to chemicals	Chemical	1.80	1.72	2	1.84
15	H17	The presence of staff not trained in waste management practices, management in case of blood and body fluid exposures	Biological	0.68	1.72	3	1.80
16	H7	Non-availability of MSDS with the staff	Chemical	1.73	1.67	2	1.80
17	H33	Non-availability of safety grab bars or side rails in toilets, corridors, and staircases	Safety	2.00	0.39	3	1.79
18	H18	Prolong working hours in a constant posture	Ergonomic	1.31	1.72	2	1.67
19	H14	Non-availability of proper storage and transportation of food items	Biological	1.50	1.42	2	1.64
20	H31	Non-functional safety alarms for medical gas supply system	Safety	2.43	0.36	2	1.59
21	H2	Inadequate illumination in the area	Physical	1.95	0.75	2	1.56
22	H15	Presence of would growth at air conditioning ducts, on walls, ceilings, shelves, etc.	Biological	1.56	1.69	1	1.41
23	H34	Non-availability of asset coding system	Safety	1.50	0.72	2	1.40
24	H38	Non-availability of security personnel at patient care units	Safety	2.00	1.21	1	1.40
25	H32	The presence of loose false ceilings, broken tiles, doors, and windows	Safety	1.63	0.45	2	1.36
26	H9	Non-availability of SOP on spill management, personal protective equipment usage, and accidental exposure to chemicals	Chemical	1.50	1.27	1	1.25
27	H13	Non-availability of pest control system	Biological	1.63	1.09	1	1.24
28	H22	Lack of awareness of employee grievance mechanism	Psychosocial	1.31	1.36	1	1.22
29	H28	Presence of loose wires, naked wires, damaged plugs, sockets	Safety	1.95	0.39	1	1.11
30	H10	Non-availability of PPE for handling chemicals	Chemical	0.65	1.60	1	1.08
31	H29	Non-availability of insulating mats and PPE in electrical rooms	Safety	1.68	0.42	1	1.03
32	H19	Non-availability of centralized collection and distribution system in hospital laboratories samples and reports, pharmacy and stores items, CSSD and oxygen cylinders (MGPS)	Ergonomic	1.25	1.66	0	0.97
33	H20	Non-availability of trolleys for shifting of materials	Ergonomic	1.43	0.45	1	0.96
34	H30	Absence of standardized color-coding of medical gas pipelines	Safety	2.10	0.39	0	0.83
35	H4	Non-availability of radiation protection and monitoring devices in the radiation exposure areas	Physical	1.71	0.57	0	0.76
36	H3	Non-availability of hearing protection devices in loud noise areas	Physical	1.13	0.09	1	0.73
37	H6	Lack of labeling of chemicals and caution signage not installed	Chemical	1.08	1.12	0	0.73
38	H5	Non-availability of personal protective equipment in areas of extreme temperatures	Physical	1.82	0.06	0	0.62

## Discussion

The data analysis used the final risk score, and the hazards were ranked in descending order for the prioritization of necessary corrective and preventive actions. HCWs not trained in fire safety as a hazard (Rank 1) were at the top of the list. Hospitals are places occupied with patients, have an oxygen supply, and also have many combustible substances like papers, files, registers, pharmacy items, consumable items, housekeeping items, cleaning and disinfection chemicals, electrical appliances, and equipment running for long hours, etc. The patients and their attendants may carry items like cigarettes, lighters, matchboxes, etc. that are generally prohibited at the hospital. Fire safety training helps identify the factors essential to preventing a fire incident and also ensures the safe evacuation of staff and patients in case of a fire mishap. Studies conducted on fire incidents mention the lack of fire safety protocols in hospitals as a major cause of fire accidents. The non-availability of appropriate fire extinguishers in the units, the lack of fire safety signage and evacuation maps, and the non-functioning of fire detection systems like smoke and heat detectors, including the fire alarm system, were the other hazards identified in the study.

Cyclones (Rank 2) are natural hazards, yet the occurrence of cyclones and damage to the hospital was the second on the list with a final average risk score of 2.78. Odisha has been visited by cyclones of varied intensity every year. The institution has learned over the years and taken the necessary steps to build resilience for facing such natural calamities. Cyclones cause major disruptions in healthcare services due to interruptions of electricity, water, medical gas, hospital supplies, communication system failure, etc. It has a deep impact on patients and HCWs, as their own families may have been affected while they were serving at the hospitals. Hence, building resilience to face such climatic hazards is essential.

The furniture and equipment undergo wear and tear over the period of usage. The lack of preventive maintenance (Ranks 7 and 8) for the same poses a risk of injury to HCWs and patients. Incidents of failure of critical equipment like infusion pumps, ventilators, cardiac monitors, OT equipment, etc. were confirmed by the HCWs but were never reported. The incident reporting system is therefore essential for tracking such issues and taking actions to prevent any major mishaps in the future.

The non-availability of IEC and SOP for waste management practices (Rank 9) had a risk score of 2.24. During the direct observations, the same was found to be available, but the staff were aware of it. IECs were installed in treatment or procedure rooms at the wards, OPDs, ICUs, and other areas like operation theaters. Failure to adopt universal precautions, non-compliance with the established infection control policies, and performing high-risk procedures that increase the risk of blood/body fluid exposure, such as withdrawing blood, working in the dialysis unit, administering blood, dressing surgical wounds, using needles and other sharp devices that lack safety features, the practice of recapping the used needles, incorrect BMW segregation practices, etc., are associated with risk. These are common in any healthcare setting but are practiced mainly by junior staff like trainee nurses, interns, and newly recruited staff. Wicker et al. [4] found that NSIs are avoidable, and preventive measures like training and the availability of IEC materials with SOP can help in reducing such incidents.

Musculoskeletal injuries are the leading cause of morbidity and disability in the healthcare workforce but are seldom reported. Darius et al. [5] identified that musculoskeletal disorders are a common occupational injury that is common in Asian nurses. The non-availability of patient shifting/transfer aids (Rank 10) like slide boards and roll-on devices was identified as a hazard. Patient transfer is a common activity in hospitals. Patients on life support devices, operative and postoperative cases, obese patients, patients on hemodialysis, etc. are generally non-ambulatory and need transfer from stretcher to patient beds or OT tables, and vice versa. During this act, several HCWs have reported sustaining injuries and developing musculoskeletal illnesses.

A lack of periodic water filter cleaning mechanisms (Rank 11) was identified in many areas. Periodic cleaning was not evident from the documents and staff interviews. Several complaints have been received from the wards, ICUs, and OPDs. It contributes to hospital-acquired infections (HAIs) and also results in staff illness. About 90% of the study participants linked it to HAIs.

Spill kits are necessary for managing accidental spillage of chemicals or blood and body fluids. It helps in clearing the spillage in a standardized manner and prevents exposure to chemicals or blood fluids. The presence of untrained staff in handling the spill makes them vulnerable to such hazards.

Though the management of chemicals in the study institution is regulated, the non-availability of material safety data sheets is identified as a hazard (H7). Chemicals like disinfectants (glutaraldehyde, sodium hypochlorite, etc.) and other cleaning agents need to be stored in a secure location with appropriate signage, a material safety data sheet, and a SOP. Chronic exposure to disinfectants like glutaraldehyde was observed in endoscopy rooms, ICUs, and OTs and can have a long-term impact on staff health. Thus, such areas require adequate ventilation systems.

The non-adherence of HCWs at the catheterization lab and operation theaters to using thermoluminescent dosimeter badges for radiation exposure monitoring is a major deficiency in terms of occupational safety.

Studies suggest that biological hazards like inadequate pest control services (H13) in certain patient care areas like the patient kitchen, the non-availability of proper storage and transportation systems for food items (H14), and the presence of would growth at air conditioning ducts, walls, ceilings, shelves, etc. (H15) are commonly found in hospitals.

Among the ergonomic hazards are prolonged working hours in a constant posture (H18); non-availability of a centralized collection and distribution system in hospital laboratories for transport of samples and reports; pharmacy and store items; CSSD and oxygen cylinders (H19); and non-availability of trolleys for shifting of materials. A good asset management system improves the life of the asset. Asset tracking in the healthcare field helps in staff satisfaction with time-saving, proper handover of equipment, and efficient patient care.

The presence of loose false ceilings, broken doors, and windows is a threat to both the HCWs and the patients. Broken tiles, doors, and windows affect patient transportation and human movement and can result in injuries.

The lack of awareness about the employee grievance mechanism (H22) was identified as a psychosocial hazard. Incidents like discrimination, harassment, and bullying at the workplace are considered psychosocial hazards and affect the work efficiency of the HCWs, making them vulnerable to committing errors or sustaining injuries.

The study conducted by Valis et al. [8] in the Czech Republic focused on the application of logical and systematic methods for risk management by establishing the context for identifying and analyzing risks associated with day-to-day activities or processes. The question for hazard identification always revolves around a few questions like "What can happen and why?" "What are the consequences?" "What is the probability of future occurrence?" and "Are there any factors that mitigate the consequence of the risk or that reduce the probability of the risk?". They also mentioned the benefits of risk assessment, like how it provides necessary information to decision-makers for policy formulation and improves services for staff and patients. The root cause of a hazard and associated risk analysis help to rethink or redesign the existing processes.

Smith et al. [9] identified that musculoskeletal disorders are a common occupational injury in Asian nurses that is associated with poor ergonomics of hospital furniture. This was also evident in this study. Dressner and Kissinger [10], in their report of occupational injuries and illnesses among nurses, mentioned the incidence of overexertion, falls, trips and slips, violence, exposure to harmful substances, fire, and explosions. About 45.6% of nurses had reported an incidence of overexertion and bodily reaction; 25% reported falls, slips, or trips; 11% reported violence; and 3.3% reported exposure to harmful substances. Gestal [11] discussed the major occupational hazards in hospitals that are affecting healthcare workers and methods for preventing them. These included accidental injuries, chemical exposure, mental health, emotional stress, and assault. He emphasized staff training as an effective strategy for risk mitigation, and their study revealed a high prevalence of occupational injuries like body fluid exposures, needle stick injuries, stress, verbal abuses, etc. A similar study carried out by Kour et al. [12] in a critical care unit in a tertiary care hospital used the staff responses for risk assessment. They identified the hindrance in the movement of patients, staff, and utilities due to the obstructed corridors, malfunctioning of the biomedical equipment, and broken equipment as the major hazards. They suggested an increased frequency of safety rounds as a suitable intervention.

This study is similar to the hazard vulnerability analysis [13] tool developed by the Kaiser Permanente Group. The severity of the hazard is calculated based on the impact of the hazard (human impact, property impact, and business impact) and the preparedness of the organization. It calculates the probability with historical and predictive data. Wells [14] highlights preventive systems as a part of process safety. If the problem is identified during the preventive period, the risk can be reduced significantly with appropriate instrumentation and control. Similarly, a HIRA exercise helps identify potential failures. The findings of the study corroborated the National Disaster Management Guidelines [15] and were similar to those conducted by Ong et al. [16].

Badubi [17] studied the role of employee motivation in building a conducive environment and its associated risks, like personnel, operational, reputational, environmental, health, and financial. This highlights the importance of risk assessment from different perspectives of staff motivation, which could not be ensured in this study. Kaya et al. [18] developed a risk assessment framework for hospitals that can be integrated with HIRA exercises. This enables the identification of risks and contributes to decision-making and risk reduction. Kour et al. [12] conducted HIRA exercises in a critical care unit of a tertiary care hospital. The hazards identified were similar to those of the present study and were focused on one particular area, whereas the present study covered almost all the areas of the hospital. The present study is based on the principle of plan-do-check-act [19], a quality improvement method that can be used as a strategy for hazard identification and ensuring workplace safety.

Strengths of the study

This study is based on national and international accreditation guidelines available for hospitals, like the National Accreditation Board for Hospitals and Healthcare Establishments (Indian Accreditation Agency) Standards for Hospitals. The study was periodically reviewed by the faculty members of the department of hospital administration and external resource persons with the necessary expertise in the field of risk assessment. "Direct observation," "document verification," and "staff interview" are comprehensive methods that were reciprocated as a checklist and used for self-assessment.

Limitations

The study area was large, with the whole hospital block having 25 operation theaters, 37 wards, and 200 critical care beds. The hazards identified and risk assessment in one area may not be in another area. Thus, HIRA in individual units can be more focused and intense. The ongoing COVID-19 pandemic also limited the interactions between HCWs.

Outcomes of the study

The study enabled the identification of hazards applicable to the institute and generated awareness among HCWs about workplace safety. It is an effort to develop comprehensive tools for ensuring safety in hospitals. HIRA for large hospitals is a difficult task and involves multiple departments; thus, it is neglected. Speaking of patient safety, workplace safety is important. The study uses inputs from all categories of staff and their experiences, verifies them with available documents, and therefore can be used as a baseline for the development of HIRA tools specific to individual healthcare institutions.

Recommendations

The study recommends the development of a hazard identification and risk management program that may include the constitution of the facility inspection team, workplace feedback from HCWs, incident reporting, and a comprehensive preventive maintenance plan for buildings, furniture, and equipment.

## Conclusions

The identification of potential hazards in a large hospital was conducted in three ways: direct observation, staff interview, and review of records. It is based on national and international standards for the assessment of healthcare quality in hospitals. The ranking of the hazards based on individual risk scores helped identify the hazards requiring necessary action for risk reduction and elimination. The HIRA exercises are a practical approach that showed several areas of improvement and thus can be used in any healthcare establishment to make it safer for HCWs as well as patients.
